# A Randomized Feeding Trial of Iron-Biofortified Beans in School Children in Mexico

**DOI:** 10.3390/nu11020381

**Published:** 2019-02-12

**Authors:** Julia L. Finkelstein, Saurabh Mehta, Salvador Villalpando, Veronica Mundo-Rosas, Sarah V. Luna, Maike Rahn, Teresa Shamah-Levy, Stephen E. Beebe, Jere D. Haas

**Affiliations:** 1 Division of Nutritional Sciences, Cornell University, Ithaca, NY 14853, USA; jfinkelstein@cornell.edu (J.L.F.); smehta@cornell.edu (S.M.); svl25@cornell.edu (S.V.L.); mr42@cornell.edu (M.R.); 2 Instituto Nacional de Salud Pública, Cuernavaca 62100, Morelos, México; svillalp@insp.mx (S.V.); vmundo@insp.mx (V.M.-R.); tshamah@insp.mx (T.S.-L.); 3 Centro Internacional de Agricultura Tropical, Cali 6713, Colombia; s.beebe@cgiar.org

**Keywords:** iron, anemia, biofortification, beans, children, Mexico, international nutrition

## Abstract

Iron deficiency is a major public health problem worldwide, with the highest burden among children. The objective of this randomized efficacy feeding trial was to determine the effects of consuming iron-biofortified beans (Fe-Beans) on the iron status in children, compared to control beans (Control-Beans). A cluster-randomized trial of biofortified beans (*Phaseolus vulgaris* L.), bred to enhance iron content, was conducted over 6 months. The participants were school-aged children (*n* = 574; 5–12 years), attending 20 rural public boarding schools in the Mexican state of Oaxaca. Double-blind randomization was conducted at the school level; 20 schools were randomized to receive either Fe-Beans (*n* = 10 schools, *n* = 304 students) or Control-Beans (*n* = 10 schools, *n* = 366 students). School administrators, children, and research and laboratory staff were blinded to the intervention group. Iron status (hemoglobin (Hb), serum ferritin (SF), soluble transferrin receptor (sTfR), total body iron (TBI), inflammatory biomarkers C-reactive protein (CRP) and α-1-acid glycoprotein (AGP)), and anthropometric indices for individuals were evaluated at the enrollment and at the end of the trial. The hemoglobin concentrations were adjusted for altitude, and anemia was defined in accordance with age-specific World Health Organization (WHO) criteria (i.e., Hb <115 g/L for <12 years and Hb <120 g/L for ≥12 years). Serum ferritin concentrations were adjusted for inflammation using BRINDA methods, and iron deficiency was defined as serum ferritin at less than 15.0 µg/L. Total body iron was calculated using Cook’s equation. Mixed models were used to examine the effects of Fe-Beans on hematological outcomes, compared to Control-Beans, adjusting for the baseline indicator, with school as a random effect. An analysis was conducted in 10 schools (*n* = 269 students) in the Fe-Beans group and in 10 schools (*n* = 305 students) in the Control-Beans group that completed the follow-up. At baseline, 17.8% of the children were anemic and 11.3% were iron deficient (15.9%, BRINDA-adjusted). A total of 6.3% of children had elevated CRP (>5.0 mg/L), and 11.6% had elevated AGP (>1.0 g/L) concentrations at baseline. During the 104 days when feeding was monitored, the total mean individual iron intake from the study beans (Fe-bean group) was 504 mg (IQR: 352, 616) over 68 mean feeding days, and 295 mg (IQR: 197, 341) over 67 mean feeding days in the control group (*p* < 0.01). During the cluster-randomized efficacy trial, indicators of iron status, including hemoglobin, serum ferritin, soluble transferrin receptor, and total body iron concentrations improved from the baseline to endline (6 months) in both the intervention and control groups. However, Fe-Beans did not significantly improve the iron status indicators, compared to Control-Beans. Similarly, there were no significant effects of Fe-Beans on dichotomous outcomes, including anemia and iron deficiency, compared to Control-Beans. In this 6-month cluster-randomized efficacy trial of iron-biofortified beans in school children in Mexico, indicators of iron status improved in both the intervention and control groups. However, there were no significant effects of Fe-Beans on iron biomarkers, compared to Control-Beans. This trial was registered at clinicaltrials.gov as NCT03835377.

## 1. Introduction

Global estimates regarding the prevalence of anemia in school-aged children, defined by the World Health Organization as hemoglobin (Hb) <115 g/L for children younger than 12 years and <120 g/L for children 12 years and older [1], range between 25% and 46% [2,3]. Iron deficiency, the most common global micronutrient deficiency [4,5], is the leading cause of anemia, accounting for approximately 25–37% [6] to 50% [7] of anemia cases.

The estimated average requirement for iron in school-aged children is 4.1 for ≤12 y and 5.9 mg for >12 years per day [8,9]. In addition to an inadequate intake of iron and low bioavailability of iron, other nutritional factors, such as vitamin B_12_ and folate, and non-nutritional factors such as inflammation also contribute to the etiology of anemia and impact human health [10,11]. In Mexico, the national nutrition and health survey (2006) reported a prevalence of anemia of 16.6% [12] and iron deficiency of 17.6% (serum ferritin <12.0 µg/L), demonstrating a considerable health risk for children [13]. Other causes of anemia in children identified in this population in Mexico included folate deficiency and vitamin A deficiency [12]. 

Interventions, including micronutrient supplementation and food fortification, have improved the iron status and reduced the prevalence of anemia in some settings. However, iron deficiency remains an urgent public health problem and threat to child health and development. Young children are at a particularly high risk due to rapid growth, inadequate dietary intake, and a high risk of infection in resource-limited settings [14]. Iron deficiency has been associated with impaired cognitive function in children and long-term impairments in physical work capacity in adulthood [15,16,17]. 

One novel approach to reducing micronutrient malnutrition is to enhance the nutrient quality of the diet through the biofortification of staple crops that are already locally accepted and consumed [18]. Consequently, biofortification has been recognized by the Copenhagen Consensus of 2008 as one of the top five solutions to current global health and nutrition challenges [19]. The success and challenges of biofortification have been previously documented [20,21]. We recently reviewed the published evidence from the three randomized efficacy trials of different iron-biofortified crops, including rice in adult Filipino women [22], pearl millet in school-aged children in India [23], and beans in women of reproductive age in Rwanda [24]. The findings demonstrated improvements in serum ferritin concentrations and total body iron concentrations, with an additional potential to benefit individuals who were iron deficient at the baseline [25]. Given this limited evidence and with no studies from Latin America, more studies with diverse populations and locally relevant crops are warranted before the implementation of a potentially important public health intervention. 

In order to target at-risk populations in Latin America, the Centro Internacional de Agricultura Tropical (CIAT) in Colombia bred and biofortified a common black bean variety (*Phaseolus vulgaris* L.), the standard black bean currently consumed widely in Central America and Mexico. In Mexico, in 2006, beans were ranked highly among the most consumed foods by school-aged children nationwide, according to Encuesta Nacional de Salud y Nutrición, a nationally representative nutrition survey [26]. Biofortification has nearly doubled the iron concentration (~100 versus ~50 mg/kg) of the standard bean variety. We hypothesized that the daily consumption of iron-biofortified beans (Fe-Beans) would improve hemoglobin, serum ferritin, and total body iron in 6 months, compared to control beans (Control-Beans). In order to examine this hypothesis, we conducted the first randomized efficacy trial of iron-biofortified beans and iron status in primary school-aged children from a low-income setting in Mexico. Special consideration was applied to assess indicators of iron status with and without anemia and consider the potential impact of inflammatory markers on iron status. 

## 2. Materials and Methods 

### 2.1. Study Population

This study was conducted in boarding schools for children aged 5–12 years between January and June 2010, in the state of Oaxaca, Mexico. In this rural setting, many low-resource children between 5 and 12 years of age (predominantly indigenous *Mixe* speakers) reside at boarding schools (*albergues escolares*) for 5 days per week; they receive 3 meals per day, prepared in common kitchens with fixed daily menus, and the meals are consumed in communal dining rooms. The schools in this setting were selected based on their students’ high risk of iron deficiency, willingness to participate, and regular consumption of black beans typical to rural Mexico. Specifically, the inclusion criteria were: boarding schools for children (5–12 years), located in a rural area approximately 60 km east of the city of Oaxaca, a high prevalence of anemia (≥15.0%) on the baseline survey, and an adequate infrastructure to sustain a 6-month feeding trial. The exclusion criteria were a prevalence of anemia of less than 15% on the baseline survey and an inadequate infrastructure to sustain a 6-month feeding trial.

### 2.2. Study Design

**Figure 1:**
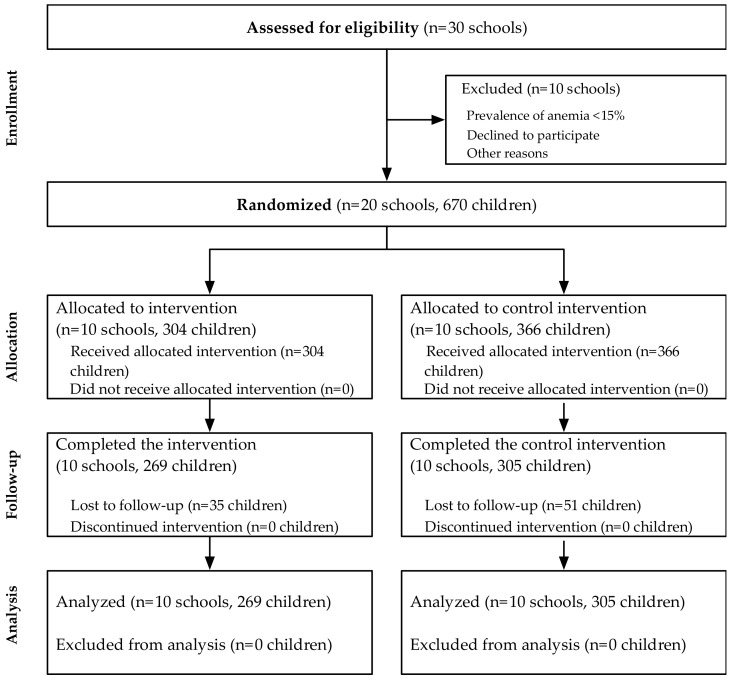
CONSORT Diagram for Cluster-Randomized Trial.

Sample selection. Boarding school administrators were contacted to explain the study design and obtain permission for prescreening. An initial prescreening survey was conducted in 30 (of 48 previously surveyed) randomly selected boarding schools, which included information on the school size, past menus, infrastructure, and capacity to support this research project (Figure 1). In all boarding schools, students provided a capillary blood sample analyzed for hemoglobin concentrations (HemoCue AB, Ängelholm, Sweden). A total of 30 schools were assessed for eligibility; 10 boarding schools were excluded based on a low prevalence of anemia in the screening survey (<15.0%) and an inadequate management and infrastructure to sustain a 6-month controlled feeding trial. The 20 boarding schools with the highest prevalence (≥15.0%) of anemia were selected; out of a total of 20 schools (*n* = 670 children), 10 were randomized to receive iron-biofortified beans (Fe-Beans; *n* = 304 children) and 10 were randomized to receive control beans (Control-Beans; *n* = 366 children) for 6 months between January and June 2010. 

Randomization and masking. The study was a double-blind, cluster-randomized controlled trial (with randomization at the school level) of: (1) iron-biofortified black beans, compared to (2) a commercial variety of beans, with the intervention randomized at the school level. The school administrators, children, research staff (for managing the intervention, food weighing, assessment of outcomes), and laboratory staff were blinded to the intervention group.

Ethics approval. Informed written assent was obtained from each participant, as well as their guardians and institution heads, at the screening and again at baseline. The Commission for Ethics, Biosafety, and Research of the Instituto Nacional de Salud Pública (INSP), Cuernavaca, Mexico, approved the research protocol.

### 2.3. Intervention

Beans: The iron-biofortified beans (*Phaseolus vulgaris* L. MIB465) were grown at the Centro Internacional de Agricultura Tropical (CIAT) in Cali, Colombia, in 2009. The beans were then shipped to Oaxaca, Mexico, for repackaging and storage. The control beans (*Phaseolus vulgaris* L. Jamapa variety) that were identical in color and size were purchased from local sources in Mexico. The two bean varieties were repackaged into knitted 50-kg plastic sacks with color-coded identifiers to distinguish the Fe-Beans and Control-Beans. The code was not known to any of the field staff, participants, or those involved in the data collection and analysis. The Fe-Beans and Control-Beans were similar in color, taste, and phytochemical content. An analysis of the iron content by inductively coupled plasma mass spectrometry (ICP-MS) before the start of the study indicated a difference of 40 μg iron per g of uncooked beans between the iron-biofortified beans (95 μg/g) and control beans (55 μg/g). All of the beans were stored in a properly ventilated and secure facility. Sacks of beans were distributed monthly to participating boarding schools from January to June 2010. 

Baseline and follow-up procedures. Individual children within boarding schools were assessed for their dietary iron intake, blood parameters, and anthropometry. The anthropometric measurements were obtained by trained research assistants using standardized procedures and calibrated instruments at the baseline and endline. Body weight was measured using a digital weighing scale (Tanita, Arlington Heights, Illinois, USA) with a precision of 100 g, calibrated daily. Height was assessed using a stadiometer (Dyna-Top, Mexico City, Mexico) with a precision of 1 mm. Individual z-scores were calculated as per World Health Organization (WHO) guidelines [27]. Mothers or housemothers of the boarding schools were asked about morbidity in children by listing symptoms related to diarrhea, upper respiratory illness, and fever. Morbidity was assessed at the baseline for 2 weeks prior to the blood sampling, as well as at endline. 

Dietary intake of iron. Children were offered 2 daily portions (100 g each) of cooked beans, one at lunch and another at dinner. The mean dietary intake for participants in this study was estimated by calculating the nutrient composition of the menus for the three meals per day offered at the boarding schools and the bean intake measured during the trial. Portion sizes were derived from the dietary test weighing performed on a monthly basis in all the boarding schools. For food weighing measurements, 1 out of 10 children were randomly selected at each boarding school in a rotation fashion. For that purpose, the portions of beans and animal-source foods (e.g., meat, eggs, cheese, or milk) were weighed before serving, and the remains were weighed again by trained personnel with OHAUS Compact Scales (Model CS, 5000), at a precision of 2 g. 

*Blood collection*. The venous blood samples (7 mL) were collected from participants for an analysis of iron status by a trained phlebotomist at the baseline and endline. Whole blood was analyzed for hemoglobin using HemoCue (HemoCue AB, Ängelholm, Sweden). Plasma was separated by centrifugation and stored below −20 °C (for < 4 days) until it was transported to the central laboratory at INSP and stored below −80 °C until analysis. Serum concentrations of ferritin and the soluble transferrin receptors (sTfR) were measured by an immunoassay method using commercial kits (Dade Behring Inc., Deerfield, IL, USA). C-reactive protein (CRP) and α-1-acid glycoprotein (AGP) were analyzed via the particle-enhanced immunoturbidometric assay in a Roche Hitachi 902 analyzer (Roche, Basel, Switzerland) at the laboratory at INSP.

Definitions of outcomes. The primary outcomes were: hemoglobin (Hb), serum ferritin (SF), and soluble transferrin receptor (sTfR) concentrations at the individual level. We also assessed total body iron (TBI), anemia, and iron deficiency. Total body iron was estimated using the approach originally proposed by Cook [28]:

TBI (mg/kg) = −[log_10_ (sTfR(mg/L) × 1000/SF(µg/L)) − 2.8229]/ 0.1207 (1)

Anemia was defined as Hb <11.5 g/dL for children younger than 12 years, and <12.0 g/dL for children 12 years and older [1]. Iron deficiency was defined as SF <15.0 µg/L for the primary analyses, and as TBI <0.0 mg/kg or sTfR >8.3 mg/L in additional analyses. 

### 2.4. Power and Sample Size Calculations

The initial power and sample size calculations were based on the following criteria: alpha (0.05), differential in iron content between the Fe-Bean and Control-Bean varieties (40 ppm), daily dry bean consumption (90 grams per day), duration of feeding (120 days of feeding), between school variance in serum ferritin (0.014 µg/L) and within school variance in serum ferritin (0.15 µg/L) concentrations. We would have an 80% power to detect a 2.7 µg/L difference in serum ferritin concentrations over the course of the study, with a sample size of 17 schools with approximately 664 children.

Statistical analysis. The descriptive statistics were expressed as the median, interquartile range (IQR) and percentages. Hemoglobin concentrations were adjusted for altitude: the hemoglobin values in the samples from children living in communities located more than 1000 m above sea level were corrected using the equation published by Cohen and Haas [29]. 

The laboratory analyses for serum ferritin concentrations for the baseline and endline were not analyzed in batch. In order to correct for significant analytical differences observed between the baseline and endline ferritin samples, a subset of samples at both time points were analyzed together. Based on these results, we developed the following equation to correct the endline ferritin values:

(2) $$\mathrm{Corrected}\;\mathrm{endline}\;\mathrm{ferritin}=\frac{\left(4.2464+\mathrm{uncorrected}\;\mathrm{endline}\;\mathrm{ferritin}\right)}{1.179782}$$

The serum ferritin concentrations were adjusted for inflammation and sex, using the BRINDA method [30], and using methods previously described [31,32,33]. The variables were natural logarithm-transformed in order to achieve normality prior to the analyses. Mixed models were used to examine the effects of Fe-Beans on hematological outcomes, compared to Control-Beans, with school as a random effect. All the models were also adjusted for the baseline value of the respective iron status indicator. *p*-values of less than 0.05 were considered statistically significant. The statistical analyses were conducted with SAS 9.4 (SAS Institute Inc., Cary, NC, USA). This trial was registered at clinicaltrials.gov as NCT03835377.

## 3. Results

### 3.1. Baseline Characteristics

Recruitment began in January 2010, and the feeding trial was conducted from January 2010 to June 2010. The baseline characteristics of children attending the 20 schools included in these analyses are presented in Table 1. After screening, 10 schools were excluded based on a low prevalence of anemia; 20 schools were randomized to receive either Fe-Beans (*n* = 10 schools; *n* = 304 children) or Control-Beans (*n* = 10 schools; *n* = 366 children) (Figure 1). Iron biomarker data were available at the endline for a total of 574 children attending 20 schools: 269 children from 10 schools received iron-biofortified beans, and 305 children from 10 schools received control beans. The median age of participants was 9.6 years (Inter-Quartile Range (IQR): 8.1, 10.9 years); 53.3% of the sample were female. There were no significant differences at the baseline between schools in treatment groups in terms of sociodemographic or anthropometric indicators, and there were no significant differences between schools or children initially enrolled (*n* = 20 schools, *n* = 670 children) and those who completed the intervention (*n* = 20 schools, *n* = 574 children). The a priori assumptions for adequate power were not met, as the number of feeding days was lower than initially anticipated (median number of feeding days was 68 vs. 120 days in power and sample size calculations). 

At the baseline, 17.8% of children were anemic; the median hemoglobin concentration was 12.8 g/dL (IQR: 11.8, 13.8 g/dL). A total of 11.3% of children were iron deficient (SF <15.0 µg/L, unadjusted), and the median unadjusted ferritin concentration was 28.7 µg/L (20.4, 38.9 µg/L). The prevalence of inflammation was 6.3% based on CRP (>5.0 mg/L) and 11.6% based on AGP (>1.0 g/L) in the overall sample. However, the prevalence of inflammation (CRP >5.0 mg/L: 8.3% vs. 4.1%; AGP >1.0 g/L: 16.1% vs. 6.4%) and morbidity (i.e., fever, diarrhea, productive cough: 38% vs. 22%) was higher in schools in the Control-Bean group at the baseline, compared to the Fe-Bean group. 

After adjusting baseline serum ferritin concentrations for inflammation (using the BRINDA method [30]), the median SF concentration was reduced from 28.7 (IQR: 20.4, 38.9 µg/L) to 24.5 (IQR: 18.2, 33.8 µg/L). The prevalence of iron deficiency at the baseline was 15.9%, after adjusting serum ferritin concentrations for inflammation. The median sTfR concentration of 4.3 mg/L was not affected by inflammation. The unadjusted median value of 5.4 mg/kg for baseline TBI was reduced to 4.8 mg/kg after the BRINDA adjustment. 

### 3.2. Effects of the Intervention on Iron Status

The effects of iron-biofortified beans on the iron status in children compared to control beans are presented in Table 2. All the analyses presented show the effects of the intervention on the iron status indicator at the endline, adjusted for the baseline indicator, with school as a random effect. The iron status indicators, including Hb, SF, sTfR, and TBI, improved in both the intervention and control groups during the 6-month trial. However, the Fe-Beans did not significantly improve the iron biomarkers (Hb, SF, TBI), compared to the Control-Beans. The soluble transferrin receptor (sTfR) values were lower in the Fe-Bean group compared to Control-Beans at the endline (*p* = 0.054). At endline, the median hemoglobin level was 13.0 (IQR: 12.2, 13.8 g/dL) in the Control-Bean group, compared to 12.9 (IQR: 11.9, 13.6 g/dL) in the Fe-Bean group. There was a significant difference in serum ferritin (*p* = 0.04) and CRP (*p* = 0.03) concentrations between the groups at the endline. However, after adjusting for inflammation using BRINDA methods, there were no statistically significant differences between the groups with regard to the serum ferritin concentrations. Similarly, compared to Control-Beans, there were no significant Fe-Bean effects on dichotomous outcomes, including anemia (<12 years: Hb <11.5 g/dL; ≥12 years: Hb <12.0 g/dL)), iron deficiency (SF <15.0 μg/L; TBI <0.0 mg/kg; sTfR >8.3 mg/L), or inflammation (CRP >5.0 mg/L; AGP >1.0 g/L).

### 3.3. Plausability Analyses

The findings from the secondary plausibility analyses of the effects of the total consumed iron from beans on the iron status are presented in Table S1 (Online Supplementary Materials). There were no significant effects of the iron consumed from beans on the iron status biomarkers.

## 4. Discussion

Iron-biofortified beans did not significantly improve iron status in school-aged children, compared to control beans in this cluster-randomized trial in Mexico. After BRINDA adjustments for inflammation, there were no significant differences between the intervention and control groups at the endline in hemoglobin, serum ferritin, or total body iron concentrations. The intervention lowered the sTfR concentrations, a marker typically not expected to be affected by inflammation, although this did not achieve any statistical significance (*p* = 0.054). Our initial power calculations were based on 120 total feeding days; however, the median number of actual feeding days achieved was only 68. 

To our knowledge, this is the first randomized controlled trial of biofortified black beans that examined their efficacy in improving iron status in children. In this biofortified efficacy trial, it was not possible to include a true placebo, which constitutes a study limitation. Iron-biofortified beans with 95 μg/g of iron were compared to control beans containing 55 μg/g of iron, rendering a difference in iron intake of 40 μg iron per g of uncooked beans. Children received a median total of 503.6 mg iron in the Fe-Bean group, compared to a total of 295.1 mg iron in the Control-Bean group, over the course of a 6-month intervention (i.e., 180 days between the baseline and endline blood collection vs. 104 total feeding days vs. a median of 68 days of bean consumption)–which would be equivalent to 2.80 and 1.64 mg/d, respectively, over 6 months. Since the estimated average requirement for iron in school-aged children ≤12 y is between 4.1 and 5.9 mg/day [8], the Fe-Bean group received approximately 47% to 68% of their daily physiological iron requirement from experimental beans while the control group received 28% to 40% of these requirements. It is expected that during the days when experimental beans were not consumed there was still some bean consumption through the children’s usual diets at home. This usual dietary consumption was not measured but assumed to be similar between randomized intervention groups. The difference in iron ingested from experimental beans between the intervention groups (i.e., 208.5 mg or approximately 1.16 mg/day) is substantial. This comparison, however, assumes that the school-aged children regularly consume the allotted two-per-day portions of beans over the entire follow-up period. Considering the anticipated low iron absorption of approximately 5% from beans [34], the net effect of the transfer of absorbed iron from the two types of beans to the iron stores and functional pools may have been inadequate to result in any significant differences between the intervention groups in the measured biomarkers. It is important to note that the improvement of the iron status in both groups indicates the baseline potential for benefits to this population, even from consumption of standard black beans. 

Other studies with biofortified crops on school-age children include one from India using biofortified pearl millet [23]. In that study, iron-biofortified pearl millet significantly improved the iron status in secondary school children after 4 months compared with control pearl millet. Although the amount of iron in biofortified pearl millet is similar to that in biofortified black beans, there are a few differences that may explain the discrepant findings. First, the amount of pearl millet consumed was far greater than the amount of beans consumed in this study; second, the bioavailability of iron in pearl millet is estimated to be higher (7.3%) [35] compared to that of black beans (5%); third, the prevalence of inflammation was considerably lower in the location in India; fourth, phytates and polyphenols may reduce iron absorption; and fifth, there was no assessment of the midline iron status in the trial in Mexico. In the study from India, one of the key findings was that although the serum ferritin concentrations were similar by the end of the trial (6 months) in both groups, there were significant benefits of biofortification at the midpoint assessment; i.e., the intervention group reached those levels significantly faster than the control group. 

The results from this trial are also inconsistent with other trials of iron-biofortified crops. With the assessment of multiple iron biomarkers, previous randomized trials have demonstrated the efficacy of the iron biofortification of other staple crops, including beans in women of reproductive age attending university, in improving the iron status in at-risk populations [22,23,24]. A meta-analysis synthesizing the evidence from these three trials found that iron biofortification interventions significantly increased serum ferritin concentrations and total body iron, compared to conventional crops, with the most substantial benefits among those who were iron deficient at the baseline [25]. 

In this study, there was a higher prevalence of elevated AGP and CRP in the control group at baseline, compared to the Fe-Bean group. Adjusting for inflammation sufficiently attenuated the high ferritin values in the control group—consistent with the impact of inflammation on the iron status—effectively reversing the apparent negative intervention effect (Table 2). A major strength of this study was the comprehensive assessment of the iron status, including iron biomarkers and indicators of inflammation (i.e., CRP and AGP). This is consistent with WHO recommendations for iron status assessment in populations, namely the examination of Hb, SF, sTfR, and at least 1 acute phase protein (i.e., CRP or AGP) [36]. Soluble transferrin receptor is a carrier protein required for iron endocytosis and regulated in response to intracellular iron levels, and it is less sensitive to inflammation or to the acute phase response in infections. Therefore, measurements of both SF and sTfR can inform the distinction between the effects of inflammation on the host iron status and changes in the iron status. It should be noted that, in this study, Fe-Beans reduced the sTfR concentrations compared to Control-Beans (*p* = 0.054), supporting the predicted effect of iron-biofortified beans improving the iron status, as this indicator is not expected to be influenced by inflammation. 

Another strength of this study was the use of black beans in a population with high baseline intake of this staple crop. In southern Mexico, the mean daily consumption of standard beans for school-aged children is, on average, 50 g of dry weight per day, which is a good iron source and can provide approximately 50% of the daily estimated average requirement of iron. This affirms the use of the black bean as an appropriate carrier for an amplified iron delivery [26].

This study has several limitations. Randomization was implemented at the school level rather than at the individual level, limiting the power of the study. The a priori assumptions for adequate power were not met, as the number of feeding days was lower than that initially anticipated (i.e., the median number of feeding days was 68 vs. the 120 days included in the power and sample size calculations). The school-level randomization also resulted in significant differences between the treatments at the baseline in inflammation, which impacts the iron assessment since ferritin is an acute-phase protein. Children in schools in the control group were significantly more likely to exhibit elevated inflammation (CRP >5 mg/L, AGP >1 g/L) at the baseline, compared to children in boarding schools in the intervention group. As part of the immune response, inflammation is associated with a variety of human disorders and is common on both ends of the spectrum of malnutrition, including obesity and undernutrition [37]. Inflammation is also common in settings with high burdens of infectious diseases. There is an established relationship between iron and inflammation [38]: inflammatory cytokines activate the secretion of the hepatic peptide hormone hepcidin to reduce the circulation of iron, thus increasing serum ferritin and decreasing transferrin-bound iron [37,39]. This can cause an anemia of inflammation, which is typically unresponsive to iron interventions. 

The indicators most susceptible to inflammation, serum ferritin and hemoglobin, responded predictably by exhibiting greater increases in the control group, which included a significantly greater proportion of children with chronic, low-grade inflammation. Additionally, the low bioavailability of iron from the high phytate diets (mostly from maize) is a limitation for both groups and may be one of several factors that reduced the likelihood of an improvement in the iron status. 

Generalizability must also be considered; the boarding school setting, with relatively low prevalence of anemia (<20%) and high bean intake, may constrain the generalizability of the intervention efficacy to other populations. Future studies should consider other subgroups at risk of iron deficiency, including pregnant and lactating women, as well as broader populations at large. 

Overall, as a plant-based, locally consumed source of iron and protein, beans offer an environmentally and economically sustainable approach for delivering nutrients in resource-limited settings, and both control and intervention groups demonstrated an improved iron status in this trial. The present study highlighted the importance of examining inflammation as part of a comprehensive iron status assessment, and adjusting for inflammation. In prospective studies, additional methods are needed to integrate changes in inflammation in the assessment of the iron status over time. Future investigations should assess the effects of iron-biofortified beans on the iron status in at-risk populations and elucidate the association of iron status and inflammatory markers in children with multiple measurements during follow-ups (e.g., random serial sampling). Future randomized efficacy trials are also needed to evaluate the effects of iron-biofortified beans on functional outcomes of iron deficiency, including cognitive development, growth, and physical performance.

## Figures and Tables

**Table 1:** Baseline characteristics of the study population and intervention.

Study Population ^1^	Total (*n =* 574)	Fe-Beans (*n* = 269)	Control-Beans (*n* = 305)
	Median ± IQR/*n* (%)	Median ± IQR/*n* (%)	Median ± IQR/*n* (%)
Age, years	9.57 (8.08, 10.89)	9.46 (8.06, 10.67)	9.67 (8.09, 11.04)
Girls, *n* (%)	306 (53.31)	134 (49.81)	172 (56.39)
Weight, kg	25.90 (22.10, 30.70)	25.50 (21.80, 30.50)	26.35 (22.50, 31.00)
Height, cm	124.90 (118.00, 132.55)	124.70 (117.60, 132.35)	125.30 (118.40, 132.85)
Body Mass Index (BMI), kg/m^2^	16.63 (15.73, 17.66)	16.36 (15.49, 17.53)	16.77 (15.96, 17.78)
BMI z-score (BMIZ)	0.16 (−0.30, 0.65)	0.11 (−0.38, 0.65)	0.20 (−0.20, 0.65)
BMIZ <−2	2 (0.35)	1 (0.37)	1 (0.33)
**Biomarkers**			
Hemoglobin ^2^ g/dL	12.82 (11.84, 13.83)	12.89 (11.89, 14.09)	12.74 (11.74, 13.64)
Anemic	102 (17.77)	47 (17.47)	55 (18.03)
Serum ferritin ^4^ µg/L	28.70 (20.40, 38.90)	28.00 (19.00, 38.10)	29.20 (21.80, 39.50)
<15.0 µg/L	65 (11.32)	40 (14.87) ^3^	25 (8.20) ^3^
<20.0 µg/L	138 (24.04)	77 (28.62)	61 (20.00)
<30.0 µg/L	309 (53.83)	147 (54.65)	162 (53.11)
Serum ferritin^4^ (BRINDA adjusted), µg/L	24.51 (18.18, 33.80)	24.20 (15.92, 32.12)	25.10 (18.58, 35.61)
<15.0 µg/L	90 (15.90)	58 (22.05) ^3^	32 (10.56) ^3^
<20.0 µg/L	182 (32.16)	97 (36.88)	85 (28.05)
<30.0 µg/L	383 (67.67)	184 (69.96)	199 (65.68)
sTfR ^4^ (Ramco corrected), mg/L	4.31 (3.99, 4.68)	4.33 (4.04, 4.69)	4.31 (3.97, 4.65)
>8.3 mg/L	0 (0.00)	0 (0.00)	0 (0.00)
Total body iron, mg/kg	5.37 (4.14, 6.51)	5.25 (3.80, 6.42)	5.54 (4.40, 6.61)
<0 mg/kg	4 (0.70)	4 (1.49)	0 (0.00)
Total body iron (BRINDA adjusted), mg/kg	4.79 (3.63, 5.97)	4.63 (3.20, 5.77)	4.94 (3.86, 6.11)
<0 mg/kg	5 (0.88)	5 (1.90)	0 (0.00)
CRP ^4^ mg/L	0.37 (0.17, 0.99)	0.38 (0.18, 1.04)	0.35 (0.15, 0.80)
>5 mg/L	36 (6.32)	11 (4.12) ^3^	25 (8.25) ^3^
AGP ^4^ g/L	0.70 (0.56, 0.84)	0.69 (0.56, 0.80)	0.71 (0.57, 0.89)
>1 g/L	66 (11.58)	17 (6.42) ^3^	49 (16.07) ^3^
**Intervention**			
Iron concentration in experimental beans, µg/g	-	94	54
Maximum potential number of feeding days	104.00 (100.00, 108.00)	104.00 (100.00, 104.00)	104.00 (100.00, 109.00)
Actual number of feeding days	68.00 (52.00, 75.00)	68.00 (51.00, 75.00)	67.00 (54.00, 75.00)
Total beans experimentally consumed, g	11965.00 (8042.00, 14090.00)	11786.00 (8227.00, 14405.00)	12022.50 (8006.50, 13876.00)
Total iron intake from dry experimental beans, mg	337.42 (256.28, 493.20)	503.58 (351.52, 615.49)	295.10 (196.52, 340.59)
Total iron absorbed from dry beans (5.0%), mg	16.87 (12.81, 24.66)	25.18 (17.58, 30.77)	14.75 (9.83, 17.03)

^1^ Median (IQR) for continuous data or n (%) for categorical data; ^2^ Hemoglobin was adjusted for altitude; anemia was defined as Hb <11.5 g/dL for <12 y and Hb <12.0 g/dL for ≥12 y. ^3^ Comparison between randomization arms at the baseline, school adjusted as a random effect; *p* < 0.05; ^4^ Natural logarithm-transformed to improve normality (results presented are not back-transformed); Abbreviations used: AGP, α-1-acid glycoprotein; CRP (C-Reactive Protein); IQR, interquartile range.

**Table 2:** Effects of consuming iron-biofortified bean on the iron status in children, compared to control beans (*n* = 574).

		Fe-Beans		Control-Beans	Intervention	
Observed Outcomes at the Endline	*n*	Median ± IQR/*n* (%)	N	Median ± IQR/*n* (%)	β (SE) or RR (95% CI)	*p*-value ^1^
Hemoglobin ^2^ g/dL	269	12.89 (11.92, 13.59)	305	13.00 (12.20, 13.84)	−0.18 (0.26)	0.50
Anemic		41 (15.24)		39 (12.79)	1.19 (0.54, 2.60)	0.66
Serum ferritin ^3^ µg/L	269	30.55 (23.26, 39.62)	305	35.55 (26.23, 48.44)	−0.17 (0.08)	0.04
<15.0 µg/L		16 (5.95)		4 (1.31)	3.42 (1.07, 10.97)	0.04
<20.0 µg/L		43 (15.99)		23 (7.54)	1.88 (0.97, 3.62)	0.06
<30.0 µg/L		129 (47.96)		117 (38.36)	1.24 (0.88, 1.74)	0.21
Serum ferritin (BRINDA) ^3^ µg/L	267	29.03 (22.12, 37.00)	305	33.13 (24.04, 43.18)	−0.10 (0.07)	0.17
<15.0 µg/L		20 (7.49)		7 (2.30)	2.32 (0.92, 5.83)	0.07
<20.0 µg/L		48 (17.98)		37 (12.13)	1.30 (0.75, 2.26)	0.35
<30.0 µg/L		143 (53.56)		134 (43.93)	1.15 (0.86, 1.53)	0.35
sTfR (Ramco corrected) ^3^ mg/L	269	4.28 (4.02, 4.57)	305	4.42 (4.06, 4.89)	−0.05 (0.02)	0.05
Total body iron, mg/kg	269	5.62 (4.64, 6.54)	305	6.00 (5.07, 7.08)	−0.43 (0.21)	0.05
Total body iron (BRINDA), mg/kg	267	5.40 (4.48, 6.32)	305	5.61 (4.79, 6.65)	−0.18 (0.18)	0.34
CRP ^3^ mg/L	268	0.27 (0.10, 0.63)	305	0.36 (0.10, 0.90)	–0.33 (0.13)	0.03
>5.0 mg/L		10 (3.73)		18 (5.90)	0.60 (0.27, 1.32)	0.20
AGP ^3^ g/L	268	0.70 (0.62, 0.81)	305	0.66 (0.57, 0.84)	0.05 (0.03)	0.12
>1.0 g/L		18 (6.72)		41 (13.44)	0.51 (0.25, 1.03)	0.06

^1^ Effects of the intervention on the endline outcome, adjusted for the baseline indicator, with school as a random effect. Generalized linear mixed models were used to examine the effects of Fe-Beans on hematological outcomes, with school as a random effect. ^2^ Hemoglobin was adjusted for altitude; anemia was defined as Hb <11.5 g/dL for <12 years and Hb <12.0 g/dL for ≥12 years. ^3^ Natural logarithm-transformation was used in the model to improve normality (results presented are not back-transformed). Abbreviations used: AGP, α-1-acid glycoprotein; CRP (C-Reactive Protein); SE, standard error; IQR, interquartile range; RR, risk ratio; 95% CI, 95 percent confidence interval.

## Supplementary Materials

The following are available online at http://www.mdpi.com/2072-6643/11/2/381/s1, Table S1: Effect of total iron consumed on iron status in children, adjusted for intervention (*n* = 571).
