# Supplementary material for: A Randomized Feeding Trial of Iron-Biofortified Beans in School Children in Mexico
Source: Nutrients. 2019 Feb 12;11(2):381. doi: 10.3390/nu11020381 (PMC6412428; doi:10.3390/nu11020381)
Supplement: Supplementary file 1 [file nutrients-11-00381-s001.pdf]

Online Supplemental Material

Table I. Effect of total iron consumed on iron status in children, adjusted for intervention (*n* = 571).

**Observed outcomes at endline**	*n*	**Effect of total iron consumed[1]** β (SE) or RR (95% CI)	***p*-value[2]**
Hemoglobin,[3] g/dL	571	-0.42 (0.48)	0.39
Anemic		3.40 (0.53, 21.94)	0.20
Serum ferritin,[4] μg/L	571	0.04 (0.14)	0.77
<15.0 μg/L		1.25 (0.06, 24.71)	0.88
<20.0 μg/L		1.78 (0.34, 9.31)	0.50
<30.0 μg/L		0.52 (0.22, 1.22)	0.13
Serum ferritin (BRINDA),[4] μg/L	561	0.04 (0.13)	0.76
<15.0 μg/L		1.92 (0.14, 27.17)	0.63
<20.0 μg/L		0.62 (0.18, 2.11)	0.45
<30.0 μg/L		0.67 (0.32, 1.41)	0.29
sTfR (Ramco corrected),[4] mg/L	571	0.00 (0.04)	0.94
Total body iron, mg/kg	571	0.16 (0.48)	0.73
Total body iron (BRINDA), mg/kg	561	0.14 (0.45)	0.76
CRP,[4] mg/L	566	-0.33 (0.42)	0.43
>5.0 mg/L		0.66 (0.04, 10.46)	0.77
AGP,[4] g/L	566	-0.09 (0.09)	0.31
>1.0 g/L		0.12 (0.02, 0.86)	0.03

[1] For every 1g of iron consumed. [2] Effects of iron consumed from beans on endline outcome, adjusted for baseline indicator and intervention, with school as a random effect. Generalized linear mixed models were used to examine the effect of total iron consumed on hematological outcomes, with school as a random effect. [3] Hemoglobin was adjusted for altitude. Abbreviations used: AGP, α-1-acid glycoprotein; CRP (C-Reactive Protein)
